# Molecular Insights into Function and Competitive Inhibition of *Pseudomonas aeruginosa* Multiple Virulence Factor Regulator

**DOI:** 10.1128/mBio.02158-17

**Published:** 2018-01-16

**Authors:** Tomoe Kitao, Francois Lepine, Seda Babloudi, Frederick Walte, Stefan Steinbacher, Klaus Maskos, Michael Blaesse, Michele Negri, Michael Pucci, Bob Zahler, Antonio Felici, Laurence G. Rahme

**Affiliations:** a Department of Surgery, Massachusetts General Hospital, Boston, Massachusetts, USA; b Department of Microbiology and Immunobiology, Harvard Medical School, Boston, Massachusetts, USA; c Shriners Hospitals for Children Boston, Boston, Massachusetts, USA; d INRS, Institut Armand Frappier, Laval, Québec, Canada; e Proteros Biostructures, Planegg-Martinsried, Germany; f Microbiology Unit, Aptuit Centre of Drug Discovery and Development, Verona, Italy; g Spero Therapeutics, Cambridge, Massachusetts, USA; Emory University School of Medicine

**Keywords:** MvfR, PqsR, *Pseudomonas aeruginosa*, structural analysis, X-ray crystallography, antivirulence, benzamine-benzimidazole inhibitors, competitive inhibition, infection, inhibition, pathogenesis, quorum sensing

## Abstract

New approaches to antimicrobial drug discovery are urgently needed to combat intractable infections caused by multidrug-resistant (MDR) bacteria. Multiple virulence factor regulator (MvfR or PqsR), a *Pseudomonas aeruginosa* quorum sensing transcription factor, regulates functions important in both acute and persistent infections. Recently identified non-ligand-based benzamine-benzimidazole (BB) inhibitors of MvfR suppress both acute and persistent *P. aeruginosa* infections in mice without perturbing bacterial growth. Here, we elucidate the crystal structure of the MvfR ligand binding domain (LBD) in complex with one potent BB inhibitor, M64. Structural analysis indicated that M64 binds, like native ligands, to the MvfR hydrophobic cavity. A hydrogen bond and pi interaction were found to be important for MvfR-M64 affinity. Surface plasmon resonance analysis demonstrated that M64 is a competitive inhibitor of MvfR. Moreover, a protein engineering approach revealed that Gln194 and Tyr258 are critical for the interaction between MvfR and M64. Random mutagenesis of the full-length MvfR protein identified a single-amino-acid substitution, I68F, at a DNA binding linker domain that confers M64 insensitivity. In the presence of M64, I68F but not the wild-type (WT) MvfR protein retained DNA binding ability. Our findings strongly suggest that M64 promotes conformational change at the DNA binding domain of MvfR and that the I68F mutation may compensate for this change, indicating allosteric inhibition. This work provides critical new insights into the molecular mechanism of MvfR function and inhibition that could aid in the optimization of anti-MvfR compounds and improve our understanding of MvfR regulation.

## IMPORTANCE

*Pseudomonas aeruginosa* is an opportunistic Gram-negative pathogen that causes serious acute, persistent, and relapsing infections. New approaches to antimicrobial drug discovery are urgently needed to combat intractable infections caused by this pathogen. The *Pseudomonas aeruginosa* quorum sensing transcription factor MvfR regulates functions important in both acute and persistent infections. We used recently identified inhibitors of MvfR to perform structural studies and reveal important insights that would benefit the optimization of anti-MvfR compounds. Altogether, the results reported here provide critical detailed mechanistic insights into the function of MvfR domains that may benefit the optimization of the chemical, pharmacological, and safety properties of MvfR antagonist series.

## INTRODUCTION

*Pseudomonas aeruginosa* is an opportunistic Gram-negative pathogen that causes serious acute, persistent, and relapsing infections (1). It adapts easily and persists in various settings (2, 3). The effectiveness of antibiotics for eliminating *P. aeruginosa* infections, which is limited by the species’ low permeability and cell wall (4), has been further complicated by the emergence of multidrug-resistant (MDR) strains (5–7). Thus, there is an urgent need for novel approaches, including new pharmacotherapies, to treat patients with *P. aeruginosa* infections (8, 9).

Traditional anti-infective therapies target essential bacterial cellular functions, thereby applying selective pressure for antibiotic resistance, which can be generated by a variety of mutations, including mutations that affect proteins that are targeted directly by antibiotics, mutations that enhance an enzyme’s antibiotic-modifying and/or -hydrolyzing activity, or mutations that increase the expression of efflux pumps (10). Bacteria can also survive antibiotic killing as a subpopulation of dormant so-called antibiotic-tolerant/persister (AT/P) cells. AT/P cells are characterized by a suppressed metabolic state that permits them to tolerate exposure to normally lethal concentrations of antibiotics (11–13). This ability, which is not consequent to antibiotic resistance mutation, has been implicated in antibiotic treatment failures and the occurrence of latent, chronic, and relapsing infections (11–13). *P. aeruginosa* excretes a small-molecule infochemical that signals for the accumulation of AT/P cells by inducing changes that are critical for pathogen adaptation and chronic infection (14–16). Thus, looking toward the development of next-generation antimicrobial drugs, it will be important to find ways to prevent AT/P cell formation as well as to interfere with nonessential bacterial functions, including bacterial virulence pathways, such that virulence may be suppressed without applying strong selective pressure favoring MDR strain emergence (17, 18).

We have demonstrated the role of multiple virulence factor regulator (MvfR), also known as PqsR, in the formation of AT/P cells (14–16) and the regulation of various virulence functions in *P. aeruginosa* (14–17, 19–25). MvfR is a quorum sensing transcriptional regulator that regulates virulence functions critical for acute, persistent, and relapsing infections, making it a high-interest novel drug target for treatment of *P. aeruginosa* infections (20, 21). MvfR controls its own activity by upregulating the expression of genes in the *pqsABCDE* and *phnAB* operons, which encode enzymes that catalyze the biosynthesis of at least 57 distinct low-molecular-weight compounds (18, 20, 21, 24, 25), including hydroxyquinolones (HAQs) (26) and the non-HAQ molecule 2-aminoacetophenone (2-AA) (15, 22, 27, 28).

In *P. aeruginosa*, 2-AA contributes to chronic infections by silencing acute virulence functions and favoring the formation of AT/P cells by modulating the transcription of genes involved in translation (16, 22). 2-AA also modulates host immune responses through epigenetic regulation (14, 15, 19). Two of the most abundant HAQs, namely, 4-hydroxy-2-heptylquinoline (HHQ) and 3,4-dihydroxy-2-heptylquinoline (*Pseudomonas* quinolone signal [PQS]), bind and activate MvfR *in vivo*, leading to the activation of MvfR-regulated virulence factors that promote infection (18, 21, 24, 25, 29). MvfR activity correlates with HHQ and PQS synthesis and the binding of these native ligands to MvfR. Thus, an essential step of MvfR regulon activation is the binding of MvfR to the *pqsABCDE* operon promoter region, and this step is influenced by the binding of the native ligands to MvfR (24, 25). Characterization of the interactions between MvfR and its native ligands is useful for understanding the basic biology of *Pseudomonas* and providing information useful for future drug optimization.

Recently, we performed a whole-cell high-throughput screen to identify small synthetic molecules that inhibit *P. aeruginosa* infection without affecting bacterial growth and viability (29). The compounds identified share a benzamide-benzimidazole (BB) backbone, which is structurally distinct from MvfR native ligands (30). A potent BB compound, M64, was shown to reduce virulence effectively and to suppress both acute and persistent/relapsing infections in mice. M64 was also active against MDR isolates of *P. aeruginosa*. In this study, we aimed to clarify how M64 inhibits MvfR protein function and obtain critical insights into MvfR function and its inhibition.

## RESULTS AND DISCUSSION

### Crystal structure of MvfR LBD in complex with M64.

**FIG 1:**
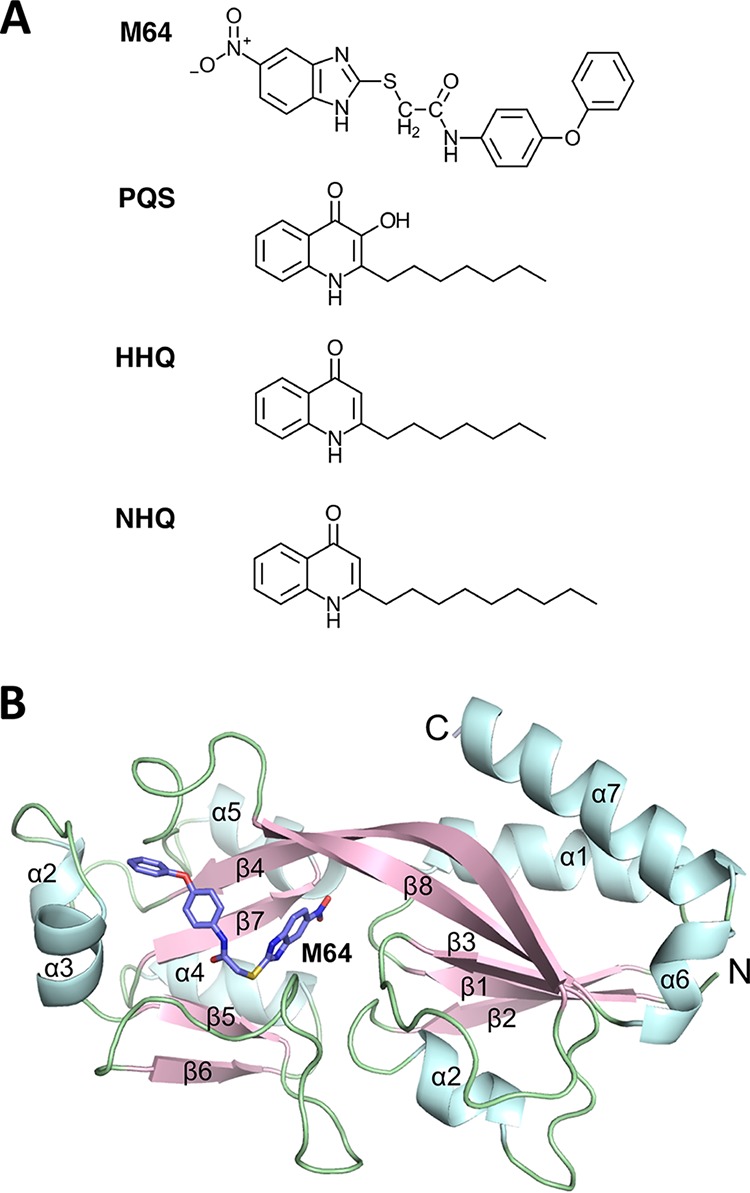
Chemical structure of M64 and crystal structure of MvfR in complex with M64. (A) Chemical structures of M64, PQS, HHQ, and NHQ. (B) Cartoon representation of the overall structure of the monomeric form of MvfR LBD (residues 93 to 295) in complex with M64. Alpha helices, beta sheets, and loops are highlighted with light blue, light pink, and light green, respectively. N, amino terminus; C, carboxyl terminus.

To clarify how M64 (Fig. 1A) interacts with MvfR, we solved the crystal structure of the MvfR ligand binding domain (LBD) complexed with M64 (MvfR^M64^) using the crystal soaking methodology under the conditions reported by Ilangovan et al. (31). The structure of MvfR^M64^ was determined in the orthorhombic space group C 2 2 2_1_ at 2.65-Å resolution, with two protein monomers in the asymmetric unit (see Table S1 in the supplemental material). The MvfR LBD consists of two subdomains connected by an antiparallel β-sheet hinge region, with a hydrophobic ligand binding pocket between the two subdomains (Fig. 1B). There are two monomers in the asymmetric unit with the same overall conformation. The model comprises residues Thr93 to Leu295. M64 was found to bind to the hydrophobic pocket where 2-nonyl-4-quinolone (NHQ) (26, 32, 33), a congener of the MvfR native ligands PQS and HHQ, also binds (Fig. 1A) (31). Although M64 has a higher molecular weight than native agonists, it is able to fit into the hydrophobic pocket owing to a bend at its central sulfur atom.

10.1128/mBio.02158-17.5 TABLE S1  Data collection and refinement statistics. Download TABLE S1, DOCX file, 0.01 MB. Copyright © 2018 Kitao et al. 2018 Kitao et al. This content is distributed under the terms of the Creative Commons Attribution 4.0 International license.

**FIG 2:**
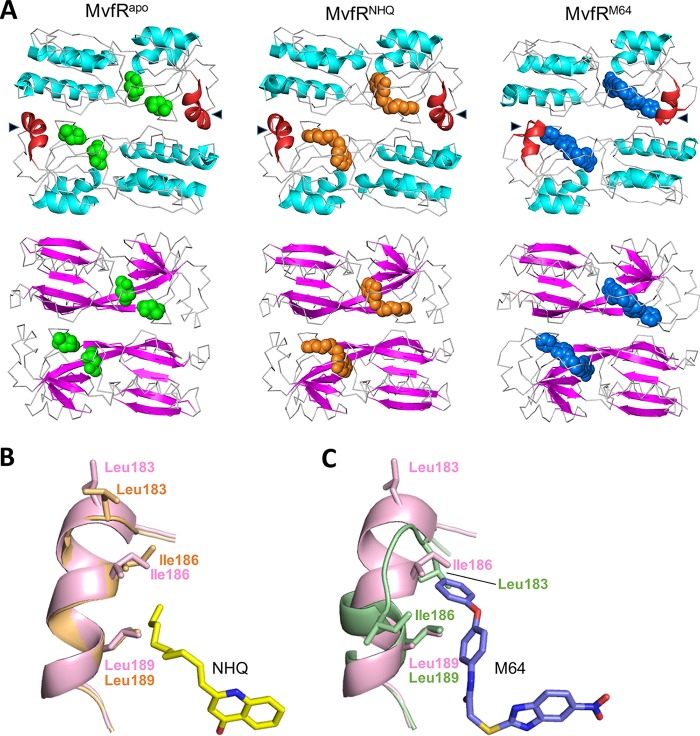
Local conformational change in M64-bound MvfR LBD. (A) Ribbon diagram of dimer form of crystal structures of MvfR LBD with no ligand (left; MvfR^apo^), NHQ (middle; MvfR^NHQ^), or M64 (right; MvfR^M64^). Alpha helices and beta sheets are indicated with cyan and pink, respectively. Green, orange, and blue spheres represent the crystallization reagent 2-methyl-2,4-pentanediol, the native MvfR ligand NHQ, and the MvfR inhibitor M64, respectively. Triangles and red alpha helices represent the residue-181-to-191 region of MvfR, which undergoes a local conformational change in the presence of M64. (B and C) A close-up view of the local conformational change. The residue-181-to-191 regions of MvfR^NHQ^ (B) (light orange) and MvfR^M64^ (C) (light green) are superimposed over MvfR^apo^ (light pink).

The overall structure of a single domain of MvfR^M64^ was compared and found to be similar to the crystal structures of both the MvfR LBD complexed with NHQ (MvfR^NHQ^) and its ligand-free form (MvfR^apo^) determined by Ilangovan et al. (Fig. 2A) (31), suggesting that there is no major conformational change in MvfR when it is bound by M64 relative to when it is bound by a native ligand (31). However, a subtle local conformational change was found in the region of helices α2 and α3 (residues 181 to 191) adjacent to the binding pocket of MvfR^M64^ (Fig. 1B and 2). As shown in Fig. 2B and C, the Leu183 residue shifts down by 3.4 Å and rotates by 180° to make direct hydrophobic contact with M64. In addition, the Ile186 residue shifts by 4.8 Å and rotates by about 90° to form a triad coordinated with Leu183 and Leu189 that retains M64 in the pocket. This conformational change was not observed in MvfR^apo^ and MvfR^NHQ^, indicating that it is specific to M64 binding. There were no local conformational differences observed in any β sheets among MvfR^apo^, MvfR^NHQ^, and MvfR^M64^.

### Hydrogen bonding and pi stacking between MvfR and inhibitor play an important role in the inhibition of the MvfR function.

**FIG 3:**
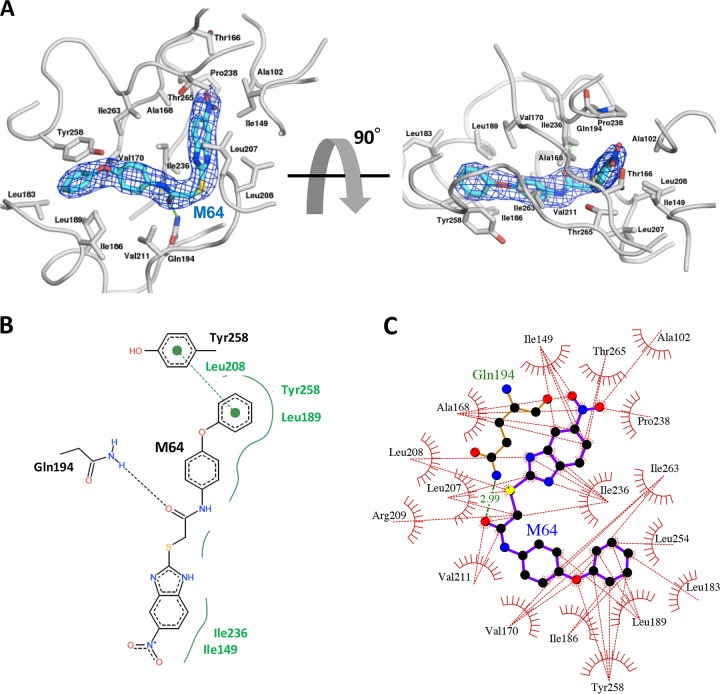
Schematic representation of M64 binding the MvfR hydrophobic pocket. (A) Diagram of the MvfR hydrophobic ligand binding pocket with M64 (blue stick). Hydrogen bonding related to Gln194 and pi-interaction-related Tyr258 are shown as green sticks. (B) Two-dimensional diagram of MvfR-M64 interaction. Black dashed line indicates hydrogen bonding. Green dashed line connecting two green dots indicates pi interaction. Green solid line indicates hydrophobic interaction surrounding M64. (C) Ligplot schematic diagram showing MvfR hydrophobic contacts with M64. Amino acids’ contributions to hydrophobic interaction between MvfR and M64 are described. Red dashed lines indicate hydrophobic interactions. Green dashed line indicates hydrogen bonding with the distance.

Figure 3A shows the binding mode of M64 to MvfR with the final 2Fo-Fc electron density map of the ligand molecule superimposed. The resulting electron density showed an unambiguous binding mode for M64 in MvfR that involves a particular orientation and conformation (Fig. 3A). Given a distance of less than 3.5 Å between donor and acceptor atoms, a specific hydrogen bond with a 2.99-Å distance between M64 and an oxygen atom in the side chain of Gln194 was identified with well-defined electron density. In addition, PoseView analysis revealed that the phenoxy group of M64 forms a pi interaction with the side chain of Tyr258 (Fig. 3B). Both the hydrogen bond and the pi interaction were not observed between MvfR and the native ligand NHQ, although they occupy the same pocket. Given our previous surface plasmon resonance (SPR) finding showing that MvfR has stronger affinity for M64 than for PQS or HHQ, the present results suggest that M64 is a highly competitive antagonist and that interaction with Gln194 and Tyr258 may enhance M64’s affinity for MvfR compared to that of NHQ, PQS, and HHQ.

A Ligplot analysis indicated that, in addition to Leu183, Ile186, and Leu189, the following residues are pivotal for generating a hydrophobic pocket in the vicinity of M64 with a maximum distance of 3.9 Å: Ala102, Ile149, Ala168, Val170, Leu207, Leu208, Arg209, Val211, Ile236, Pro238, Leu254, Ile263, and Thr265 (Fig. 3C). These hydrophobic residues are also critical for the formation of the hydrophobic pocket that mediates MvfR interaction with NHQ and 3-amino-7-chloro-2-*n*-nonyl-4(3*H*)-quinazolinone (3-NH_2_-7-Cl-C9-QZN), which is a quinazolinone (QZN) MvfR inhibitor reported in reference 31.

**FIG 4:**
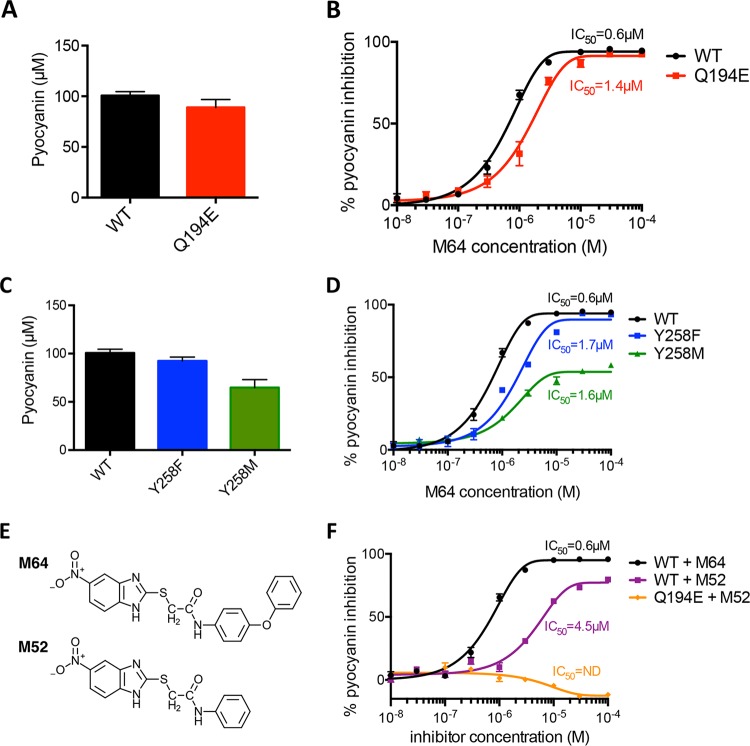
Importance of Gln194 and Tyr258 residues for MvfR inhibition. (A and C) Basal pyocyanin production of Q194E (A) and Y258 (C) mutants compared to that of WT MvfR. (B and D) M64 IC_50_ curves for Q194E (B) and Y258 (D) mutants. (E) Chemical structures of M64 and M52. (F) M64 IC_50_ curves for evaluation of the importance of pi interaction. ND, not determined.

Full-length MvfR mutants with mutations at either Gln194 or Tyr258 were constructed and transformed into the in-frame *mvfR* deletion mutant (Δ*mvfR*) to evaluate the importance of these residues for MvfR function, as indexed by pyocyanin production (Fig. 4). For the Gln194 residue, a Q194E mutant, which is theoretically unable to form a hydrogen bond with the oxygen atom of the carbonyl group in M64, was generated by replacing glutamine with glutamic acid. As shown in Fig. 4A, Q194E retained at least 88% functionality with no statistical difference in pyocyanin production compared to wild-type (WT) MvfR without M64. However, it was reported that the Q194E mutant was virtually inactive and almost failed to respond to HHQ or PQS in the *pqsA* mutant background (31); this difference could be due to the sensitivity of the readout used, since it was based on the chromosomally integrated *pqsA-lux* activity, or perhaps due to the strain used, PA14 versus PAO1. An inhibition curve showed that, like WT MvfR, Q194E could be inhibited by M64 in a dose-dependent manner (Fig. 4B). However, the half-inhibitory concentration (IC_50_) for Q194E was 2.3-fold greater than that for WT MvfR. These results suggest that although Q194 acts as the hydrogen bond donor in M64 inhibition of MvfR, this contribution is not essential for M64 inhibition of MvfR.

As for the Tyr258 residue, two mutants, Y258F and Y258M, were generated. The former has a phenylalanine substitution that retains, at least theoretically, the ability to make a pi-pi interaction with the phenoxy group in M64. The latter has a methionine substitution that renders it unable to form a pi-pi interaction with the phenoxy group in M64. Both the Y258F and Y258M mutants produced pyocyanin without M64, albeit at a slightly lower level than WT MvfR (Fig. 4C). These results and those showing that a Y258A mutation renders MvfR inactive (31) strongly suggest that a hydrophobic amino acid, especially an aromatic residue, is required at the 258th position for MvfR functionality. An inhibition curve demonstrated that Y258F could be inhibited by M64 in a dose-dependent manner, like WT MvfR, whereas Y258M could not be fully inhibited by M64 and retained weak pyocyanin production even in the presence of high concentrations of M64, indicating that M64 could not achieve full inhibition without the pi-pi interaction (Fig. 4D). The IC_50_s of Y258F and Y258M were similar to each other and 2.8- and 2.7-fold greater than the wild-type IC_50_, respectively. These results indicate that Tyr258 has a critical role in the formation of pi-pi interactions with M64 in the inhibition of MvfR function, in addition to its previously described role in native ligand recognition (31).

The importance of Gln194 and Tyr258 was also evaluated with another BB compound, M52, which is a congener of M64 that lacks the phenoxy group involved in pi-pi interaction between MvfR and M64 (Fig. 4E). As shown in Fig. 4F, M52 was unable to inhibit WT MvfR function fully and had an IC_50_ nine times higher than that of M64. Furthermore, the inhibition response was tested with a combination of the Q194E mutant and M52 to evaluate the consequences of losing both hydrogen bonding and the pi-pi interaction. M52 was unable to inhibit Q194E activity, even at high concentrations. These results demonstrate that both hydrogen bonding and pi-pi interactions have important roles in the mode of action of M64 in antagonizing the function of full-length MvfR protein.

Previously, we showed that the nitro group of BB inhibitors is important for their interaction with MvfR (30). Although specific bonding between the BB nitro group and MvfR was not seen in our crystal structure, the BB nitro group may be able to form an unstable hydrogen bond with the hydroxyl group of Thr265 in MvfR, which is adjacent to the BB nitro group in the hydrophobic pocket, in a manner similar to the interaction between Thr265 of MvfR and the 7-Cl substituent of the QZN inhibitor 3-NH_2_-7-Cl-C9-QZN (31). When a higher concentration of M52 (>30 µM) was added (Fig. 4F), pyocyanin production increased slightly, suggesting that BB compounds that lack a phenoxy group or do not form a pi-pi interaction with MvfR may become partial agonists when MvfR lacks Q194 as a hydrogen bond donor. According to the crystal structure of MvfR^NHQ^, NHQ did not form a hydrogen bond with MvfR (31). These findings suggest that, in addition to pi stacking, the formation of a hydrogen bond between MvfR and an antagonist may be important for inhibition of MvfR function. In agreement, a hydrogen bond between MvfR and QZN was found to be important for inhibition of MvfR function (31).

### M64 acts as a competitive inhibitor of MvfR.

**FIG 5:**
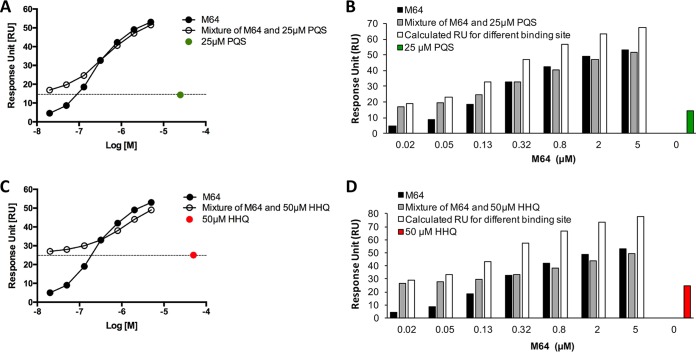
Cross-competition assay between M64 and native ligands. (A) Measured responses for M64 and PQS and a mixture of PQS and M64. Dashed line indicates measured response for PQS (25 µM). (B) Measured responses for M64 and a mixture of M64 (amount as indicated) with 25 µM PQS, calculated response units (RU) for the mixture expected for different binding sites, and measured response for 25 µM PQS alone. (C) Measured responses for M64, HHQ, and HHQ-M64 mixture. Dashed line indicates measured response for HHQ (50 µM). (D) Measured responses for M64 and mixture of M64 with 50 µM HHQ, calculated RU for the mixture expected for different binding sites, and the measured response for 50 µM HHQ alone.

We carried out a cross-competition assay with M64 and native ligands to determine whether M64 and native ligands occupy the same binding site on MvfR as was shown in the crystal structural data. We demonstrated previously in an isothermal titration calorimetry (ITC) assay that M64 binds directly to the MvfR LBD (29). However, here, we used surface plasmon resonance (SPR) analysis instead of ITC because it was difficult to maintain two hydrophobic small molecules together with the MvfR LBD protein fragment in an ITC assay. As shown in Fig. 5, the experimental responses for the mixture of M64 and PQS were increased by adding M64 incrementally to 25 µM PQS in a dose-dependent manner; the values were similar to those measured for M64 when more than 32.6 µM M64 was mixed with 25 μM PQS (Fig. 5A). The calculated response for the mixture expected for different binding pockets was higher than the experimental responses measured for the mixture of M64 with 25 μM PQS (Fig. 5B). Similarly, the experimental response for the mixture of M64 with HHQ was increased by adding M64 incrementally to 50 µM HHQ in a dose-dependent manner, and no significant differences were observed between the mixture response values and those for M64 alone when more than 32.6 µM M64 was mixed with 50 μM HHQ (Fig. 5C). The calculated responses for the mixture expected for different binding pockets were also higher than the experimental responses measured for the mixture of M64 with 50 μM HHQ (Fig. 5D). For both native ligands, the experimental response for the mixture of M64 and native ligands was higher than that measured for M64 alone when less than 32.6 µM M64 was mixed with the ligands. This difference can probably be attributed to the total concentration of M64 and ligand not reaching the maximum capacity of the total MvfR protein immobilized on the chip. Taken together, these results demonstrate that M64 binds the same hydrophobic pocket as native ligands despite their structural dissimilarity (~0.13 in ChemMine analysis) (34). These results are corroborated by the crystal structure of the MvfR-M64 complex and provide strong support for the proposition that M64 is a competitive inhibitor of MvfR.

The fact that M64 binds to the hydrophobic pocket bound by HHQ raises the question of whether M64 can also target PqsH, an enzyme that catalyzes the conversion of HHQ into PQS. To address this question, PQS production was quantified from the supernatant of *pqsA* mutant cells grown in the presence of HHQ and M64. Mutation in *pqsA* leads to a loss of HAQ production, including PQS and HHQ. As shown in Fig. S1 in the supplemental material, we observed significant PQS production in *pqsA* mutant cells grown with HHQ or both HHQ and M64. Meanwhile, no PQS production was observed in the *pqsA* mutant in the presence of only M64, demonstrating that M64 does not interfere with PqsH enzyme function.

10.1128/mBio.02158-17.1 FIG S1  M64 does not target *pqsH***.** The *pqsA* polar mutant was grown in LB for 7 h (37°C, 200 rpm) in the absence or presence of 40 µM HHQ or 10 µM M64. A 500-µl volume of culture was mixed with an equal volume of methanol containing 10 ppm of HHQ-D4 and 20 ppm of PQS-D4 and then centrifuged for 5 min at 12,000 × *g*, and then 700-µl aliquots of supernatant were transferred into glass vials. PQS levels were detected as described previously (26, 43). Download FIG S1, TIF file, 0.2 MB. Copyright © 2018 Kitao et al. 2018 Kitao et al. This content is distributed under the terms of the Creative Commons Attribution 4.0 International license.

### Nonphysiological variants insensitive to M64 aid in the understanding of MvfR activity and the functional impact of MvfR inhibition.

**FIG 6:**
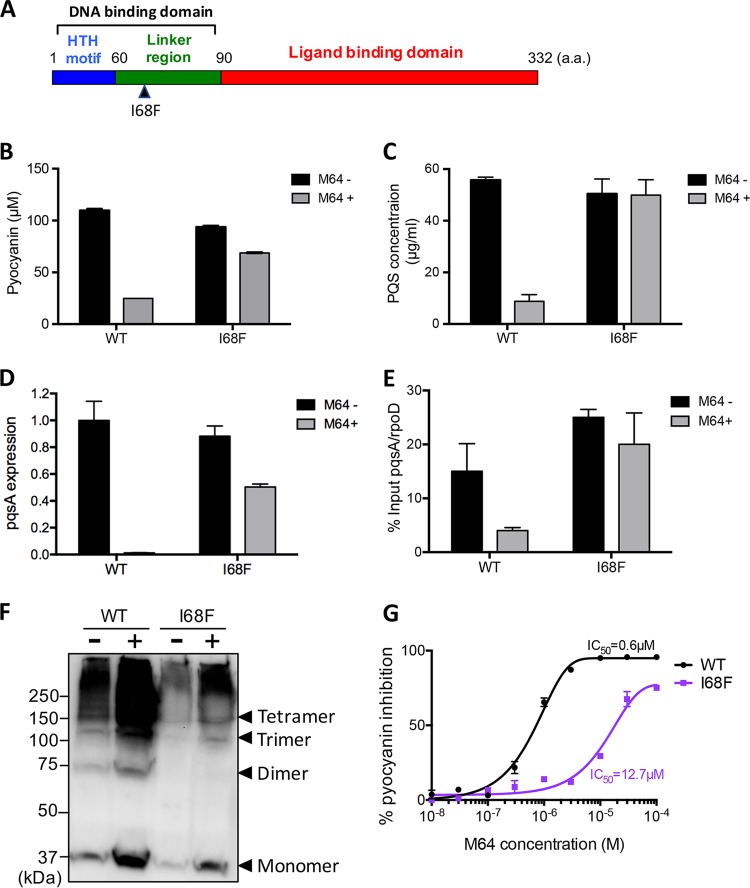
Characterization of an M64-insensitive MvfR mutant. (A) Schematic diagram of MvfR domains and the location of the I68F mutation. (B and C) Pyocyanin (B) and PQS (C) production by an I68F mutant in the absence or presence of 10 µM M64 compared to that of WT MvfR. (D and E) *pqsA* expression (D) and *pqsA* promoter binding ability (E) of the I68F mutant in the absence or presence of 10 µM M64 compared to that of WT MvfR. (F) *In vivo* cross-linking assay of WT MvfR and I68F mutant in the absence (−) and presence (+) of 10 µM M64. (G) M64 IC_50_ curve for the I68F mutant.

To clarify the mode of action of M64, we developed a system to screen for nonphysiological variants of MvfR that are M64 insensitive and/or affect MvfR’s requirement for a bound ligand to interact with promoters. The screen was based on a combination of random mutagenesis and a reporter system consisting of the *pqsA* promoter fused to the *lacZ* gene (P*pqsA-lacZ*) (Fig. S2). In this system, a plasmid encoding full-length MvfR was mutagenized in the *Escherichia coli* XL1-Red mutator strain (Agilent Technologies, Inc.) (35), and the resulting plasmids are transformed into the PA14 isogenic *ΔmvfR* in-frame deletion mutant strain with P*pqsA-lacZ* integrated into its genome. M64-insensitive MvfR variants were identified by blue/white screening in the presence of M64, as described for Fig. S2. In the first screen of more than 2,000 colonies, we obtained 38 light or dark blue colonies. The isolated blue colonies were grown in Luria-Bertani (LB) liquid medium containing 10 µM M64 to eliminate false positives for M64 insensitivity. Of the 38 isolated colonies, 3 produced pyocyanin in the presence of M64, indicating that they were M64 insensitive. The DNA sequencing results revealed that all three of the M64-insensitive colonies had an amino acid replacement of the 68th isoleucine with a phenylalanine (I68F), which is in the linker domain between the DNA binding domain and the MvfR LBD (Fig. 6A).

10.1128/mBio.02158-17.2 FIG S2  Experimental strategy for identification of M64-insensitive MvfR mutants. The screen was based on a combination of random mutagenesis and a reporter system consisting of the *pqsA* promoter fused to the *lacZ* gene. In this system, the pJN-MvfR-His plasmid was mutagenized in the XL1-Red *E. coli* mutator strain, and the resulting plasmid library was transformed into a PA14 isogenic *mvfR* mutant strain (20) containing the *pqsA*-expressing P_*pqsA*_-lacZ plasmid integrated into its genome (*mvfR*::mini-CTX-P*pqsA′*-′*lacZ*). M64-insensitive MvfR variants were identified by blue/white screening in the presence of M64. Download FIG S2, TIF file, 6 MB. Copyright © 2018 Kitao et al. 2018 Kitao et al. This content is distributed under the terms of the Creative Commons Attribution 4.0 International license.

Production of pyocyanin and PQS, which are normally inhibited by M64, is slightly affected and unaffected, respectively, by M64 in strains with the I68F substitution (Fig. 6B and C). Similarly, as assessed by real time quantitative PCR (RT-qPCR) the functionality of the I68F mutant to activate the *pqsA* promoter was retained in the presence of M64, whereas that of WT MvfR was completely impaired by M64 (Fig. 6D). Chromatin immunoprecipitation-qPCR (ChIP-qPCR) analysis to determine the DNA binding ability of the MvfR *pqsA* promoter indicated that the I68F variant of MvfR binds to the *pqsA* promoter region in the absence or presence of M64, unlike the WT protein, whose association with the *pqsA* promoter region is reduced in the presence of M64 (Fig. 6E). Overall, although not fully, the I68F mutant retains the ability to produce PQS, activate *pqsA* gene expression, and bind the *pqsA* promoter in the presence of M64, whereas these abilities are reduced radically by M64 in WT MvfR (Fig. 6B to E).

Because specific residues, such as Q194 and Y258, are important for MvfR interaction with M64, we questioned why these mutants were not identified in the screen as M64-insensitive variants. Relative to the I68F mutant, the Q194E and Y258M mutants had lower pyocyanin production in the presence of M64 (Fig. S4), suggesting that *pqsA* promoter activation by these mutants is reduced relative to that of I68F. This lesser *pqsA* promoter activation may underlie the lack of blue Q194E and Y258M mutant colonies in our screen for M64-insensitive MvfR mutants and thus may provide an explanation for Q194E and Y258M mutants not being M64-insensitive variant screen hits. Moreover, although M64 might be expected to act as an agonist of the I68F mutant, the M64 IC_50_ curve showing dose-dependent inhibition of I68F activity at high concentrations of M64 rules out that possibility (Fig. 6G). The residual inhibitory activity of M64 could be a consequence of inverse agonism, since MvfR I68F appears to have a constitutive, ligand-independent level of activity.

### MvfR inhibition by M64 does not disrupt its oligomerization.

MvfR belongs to the LysR-type transcriptional regulator (LTTR) family, which is widely conserved among prokaryotes. LTTRs are known to form regulatory multimers at target gene promoter regions. The present and prior findings demonstrated that the MvfR LBD forms a tetramer in its crystal structure in complex with inhibitors, although the association angle of two dimers in the crystal structure of MvfR-M64 complex was different from that of the MvfR-NHQ complex (Fig. S3). These data suggest that full-length MvfR may also form a tetramer complex; however, this has not yet been elucidated experimentally, and it is still questionable how the I68F mutant behaves in the absence and presence of M64. To clarify this point, an *in vivo* cross-linking assay of full-length MvfR and I68F mutant with or without M64 was carried out. Interestingly, the result showed that both WT MvfR and the I68F mutant formed multimers regardless of the presence or absence of M64 (Fig. 6F). Moreover, these results show that MvfR does indeed form an oligomer like other studied LTTRs (36). For both the MvfR WT and the I68F mutant, there were differences in the level of formation of multimers in the absence and presence of M64. These data suggest that M64 binding to MvfR protein may increase the protein stability. Altogether, these results suggest that M64 triggers a conformational change in the DNA binding domain of MvfR without promoting substantial conformational change in its LBD, consistent with allosteric inhibition. An I68F mutation at the linker domain between the helix-turn-helix (HTH) motif and the LBD may compensate for this effect without affecting MvfR oligomerization. Structural analysis using full-length MvfR, which is beyond the scope of this work, and the I68F mutant will be required for the direct demonstration of allosteric effects.

10.1128/mBio.02158-17.3 FIG S3  Tetrameric arrangement observed in the crystal structure of the MvfR-M64 and MvfR-NHQ complexes. (A) Tetrameric rearrangement of MvfR-M64 complex and its 90° angle rotated form. (B) Tetrameric rearrangement of MvfR-NHQ complex and its 90° angle rotated form from reference 31. Alpha helices and beta sheets are indicated with cyan and pink, respectively. Orange and blue spheres represent the native MvfR ligand NHQ and the MvfR inhibitor M64, respectively. Hexacobalamin molecules used as an additive for crystal soaking experiments are shown as red dots. The model was produced using PyMOL software. Download FIG S3, TIF file, 6 MB. Copyright © 2018 Kitao et al. 2018 Kitao et al. This content is distributed under the terms of the Creative Commons Attribution 4.0 International license.

10.1128/mBio.02158-17.4 FIG S4  Pyocyanin production by I68F and Y258M mutants in the presence of M64. The Δ*mvfR* mutant expressing WT MvfR, an I68F mutant, or a Y258M mutant was grown in LB containing 0.2% l-arabinose, 30 µg/ml gentamicin, and 10 µM M64 for 16 h. Pyocyanin was quantified by measuring the absorbance at 690 nm with the culture supernatants clarified by centrifugation. Download FIG S4, TIF file, 0.2 MB. Copyright © 2018 Kitao et al. 2018 Kitao et al. This content is distributed under the terms of the Creative Commons Attribution 4.0 International license.

### Conclusions.

In this study, we elucidated aspects of the molecular mechanism of MvfR function and inhibition by a nonligand BB compound. This is the first report to describe intermolecular interactions between MvfR and BB molecules, which, although structurally distinct from MvfR’s native ligands, target the same hydrophobic pocket in its LBD. Given that BB compounds may have more than one target in the MvfR-regulated quorum sensing biosynthetic pathway (30), the structural basis of the interaction between MvfR and BB inhibitors presented here provides new insights into the design of improved anti-MvfR agents that could be used to treat both acute and persistent *P. aeruginosa* infections. Moreover, the unprecedented generation of a nonphysiological variant insensitive to M64 that demonstrates a potential allosteric effect of the BB inhibitor on the MvfR DNA binding domain may also facilitate the future generation of optimized MvfR antagonists. Further structural studies with this mutant could shed light not only on how BB series inhibitors impact the conformation of full-length MvfR but also on the basic biology of LTTR protein dynamics. Altogether, the results reported here provide critical detailed mechanistic insights into the function of MvfR domains that may benefit the optimization of the chemical, pharmacological, and safety properties of MvfR antagonist series.

## MATERIALS AND METHODS

### Crystallography.

The protein construct used for crystal structure determination contained residues 94 to 295 and was preceded by His-THB-SUMO-ULP-Thr (THB, thrombin cleavage site; ULP, SEN protease cleavage site). The construct was cloned into pET28a. The protein was expressed in *E. coli* BL21(DE3) at 18°C overnight. Expression was induced at an optical density at 600 nm (OD_600_) of 0.6 with 0.1 mM isopropyl-β-d-thiogalactopyranoside (IPTG). The protein was purified by nickel-nitrilotriacetic acid (Ni-NTA) chromatography, negative Ni-NTA chromatography after cleavage of the tag with SEN protease, and a final gel filtration step. For the complex formation, the protein at a concentration of 1.54 mg/ml was incubated with 150 µM ligand M64 for 3 h at room temperature before concentration.

The protein-ligand complex in 20 mM Tris-HCl (pH 7.4), 150 mM NaCl, and 0.5 mM TCEP [tris-(2-carboxyethyl)phosphine] was crystallized at 3.3 mg/ml from 31% MPD (2-methyl-2,4-pentanediol), 90 mM imidazole (pH 8.0), 180 mM MgCl_2_, and 10 mM Co(NH_3_)_6_Cl_3_.

### Data collection and refinement.

X-ray diffraction data were collected at a temperature of 100 K at the Swiss Light Source (beamline PXI/X06SA) using a Pilatus 6M detector. Data were integrated, scaled, and merged using XDS (37). The structure was refined with REFMAC5 (38). Manual model completion was carried out using Coot (39). The final structure was obtained by molecular replacement using a previously solved structure of MvfR (PDB identifier [ID] 4JVD) as a search model. Subsequent model building and refinement were performed according to standard protocols in Coot. The quality of the final model was verified by PROCHECK (40) and the validation tools available through Coot (39).

### Construction of in-frame *mvfR* mutant.

A *P. aeruginosa ΔmvfR* in-frame deletion mutant was constructed by homologous recombination. To construct pEX18-mvfR, we amplified DNA fragments including 500 bp upstream and 500 bp downstream of MvfR using PCR with primer sets mvfR-Up-F/mvfR-Up-R and mvfR-Down-F/mvfR-Down-R (see Table S2 in the supplemental material), respectively, and then ligated them with BamHI-digested pEX18T using a Gibson assembly. The resulting plasmid was introduced into PA14 cells via conjugation, and the *ΔmvfR* mutant was generated by allelic exchange. We validated the *ΔmvfR* mutants using PCR with an mvfR-Up-F/mvfR-Down-R primer set and DNA sequencing.

10.1128/mBio.02158-17.6 TABLE S2  List of primers used in this study. Download TABLE S2, DOCX file, 0.01 MB. Copyright © 2018 Kitao et al. 2018 Kitao et al. This content is distributed under the terms of the Creative Commons Attribution 4.0 International license.

### Construction of full-length MvfR expression plasmid.

The C-terminally His-tagged full-length MvfR expression plasmid pJN-mvfR-Fx-His (Fx means Factor Xa cleavage site) was constructed as follows. The 18-bp upstream region of *mvfR* containing the Shine-Dalgarno sequence and the intact MvfR open reading frame (ORF) were amplified by PCR using primers MvfR-F and His-FactorXa-mvfR-R (Table S2) from PA14 genomic DNA. Then, the obtained PCR fragment was ligated with EcoRI-digested pJN105 plasmid vector (41) using the Gibson assembly system. The resulting plasmid sequence was analyzed at the Massachusetts General Hospital (MGH) DNA core. Finally, pJN-mvfR-Fx-His was transformed into the Δ*mvfR* mutant, and then the expression of full-length MvfR in the presence of l-arabinose protein was confirmed by Western blotting using anti-His antibody.

### Site-directed mutagenesis.

We performed site-directed mutagenesis using primer sets (listed in Table S2) with a QuikChange mutagenesis kit (Agilent Technologies, Inc.) according to the manual. The pJN-mvfR-Fx-His plasmid was used as a template. DNA sequencing of resulting plasmids was performed at the MGH DNA core. The resulting plasmids were transformed into *P. aeruginosa ΔmvfR* mutants for analysis. The protein expression level of MvfR and of the generated mutants in the presence of 0.2% l-arabinose was verified to be the same by Western blotting using anti-His antibody (data not shown).

### Cross-competition assay between M64 and native ligands by SPR.

MvfRc87 protein, 50 μg/ml in 10 mM sodium acetate buffer (pH 5.5), was covalently immobilized on a CM7 Series S sensor chip using an amine coupling reagent kit (GE Healthcare) at the range level of 3,000 to 5,000 response units (RU). Phosphate-buffered saline (PBS), pH 7.4, containing 0.05% P20 surfactant was used as the running buffer during protein immobilization. The interaction of M64 with the MvfRc87 ligand binding domain in the presence or in the absence of 25 µM PQS and 50 µM HHQ—naturally occurring ligands—was analyzed by Biacore T200 evaluation software 2.0 (GE Healthcare). SPR cross-competition binding experiments using a 2.5-fold dilution series of M64 in the presence of a fixed concentration of HHQ or PQS were performed at 25°C in 10 mM HEPES (pH 7.4), 150 mM NaCl, 3 mM EDTA, 0.05% P20 surfactant, and 4% dimethyl sulfoxide (DMSO) at a flow rate of 30 μl min^−1^ with a 60-s contact time and a 210-s dissociation time. Each injection was followed by an extra wash with 50% DMSO. Solvent correction curve cycles were also included. The relative response units (RUs) at 5 s before the end of association were extracted from the double-reference-corrected sensorgrams at different concentrations. These responses were plotted against their respective concentrations for M64 alone and in the presence of native MvfR ligand and compared to the calculated responses for the mixture expected for different binding sites.

### Determination of IC_50_.

The *ΔmvfR* deletion mutant carrying pJN-MvfR-Fx-His and its mutant expression plasmid (pJN-MvfR-Q194E-His, pJN-MvfR-Y258F, or pJN-MvfR-Y258M) was grown overnight in glass tubes containing 5 ml LB with 30 µl/ml gentamicin. The culture was diluted to an OD_600_ of 0.1 and grown with incrementally added M64 in a new glass tube containing 5 ml LB with 30 µl/ml gentamicin and 0.2% l-arabinose for 16 h at 37°C. An IC_50_ curve was plotted for percentage of pyocyanin production at each concentration of M64. The IC_50_ values were calculated using Prism software. Pyocyanin was quantified as described previously (42).

### Quantification of HAQs.

The Δ*mvfR* deletion mutant carrying pJN-MvfR-Fx-His and its mutant expression plasmid (pJN-MvfR-Q194E-His, pJN-MvfR-Y258F, or pJN-MvfR-Y258M) was grown overnight in glass tubes containing 5 ml LB with 30 µg/ml gentamicin. Bacterial cultures were diluted to an OD_600_ of 0.05 in 5 ml LB containing 30 µg/ml gentamicin and 0.2% l-arabinose with or without inhibitor in glass tubes and then grown at 37°C. When OD_600_ levels reached 3.0, 500 µl of sample was collected and mixed with an equal volume of methanol containing 20 ppm of PQS-D4 (deuterated PQS standard) and 10 ppm of HHQ-D4 (deuterated HHQ standard). After the mixture was centrifuged, 700 µl of supernatant was taken and stored in glass vials at 4°C for further liquid chromatography-mass spectrometry (LC-MS) analysis. HAQs were quantified by liquid chromatography-mass spectrometry as described in detail elsewhere (26, 43).

### Screening for M64-insensitive MvfR variants.

The MvfR expression plasmid pJN-mvfR-Fx-His, which encodes C-terminally His-tagged full-length MvfR, was transformed into the XL1-Red *E. coli* mutator strain (Agilent Technologies, Inc.). Colonies of transformants were collected and grown in LB with 30 µg/ml gentamicin, and then plasmids were extracted to make a mutated plasmid library. The 50-ng/ml amounts of mutated plasmids were transformed into Δ*mvfR* mutants carrying genomically integrated *pqsA* promoter (P*pqsA*) fused to *lacZ* (Δ*mvfR*::mini-CTX-P*pqsA′*-′*lacZ*). Transformants were selected on LB agar plates containing X-Gal (5-bromo-4-chloro-3-indolyl-β-d-galactopyranoside) and 10 µM M64. Hits were validated by DNA sequencing.

### RT-qPCR and ChIP-qPCR.

RT-qPCR to analyze *pqsA* gene expression and ChIP-qPCR to analyze the DNA binding ability of MvfR were performed as described in reference 23.

### *In vivo* cross-linking assay.

Overnight cultures of *ΔmvfR* mutants carrying pJN-mvfR-Fx-His WT or pJN-mvfR-Fx-His I68F grown in LB containing 30 µg/ml gentamicin were centrifuged, and the pellet was washed with fresh LB. Cells were diluted to an OD_600_ of 0.05 in 5 ml LB containing 30 µg/ml gentamicin with or without 10 µM M64 and grown at 37°C. Two hours later, protein expression was induced by adding 0.2% l-arabinose. When OD_600_ levels reached 2.5, bacterial cells were cross-linked with 1% formaldehyde at room temperature for 30 min, and the cross-linking reaction was quenched by adding 250 mM glycine. After washing cross-linked bacterial cells with PBS buffer, the bacterial pellet was resuspended in SDS-PAGE sample buffer, sonicated to reduce viscosity, and boiled at 65°C for 10 min. Cross-linked proteins were analyzed by Western blotting using anti-His antibody.

### Accession number(s).

Atomic coordinates and structure factors for the reported crystal structures here have been deposited with the Protein Data Bank under accession number 6B8A.
