# Supplementary material for: Molecular Insights into Function and Competitive Inhibition of Pseudomonas aeruginosa Multiple Virulence Factor Regulator
Source: mBio. 2018 Jan 16;9(1):e02158-17. doi: 10.1128/mBio.02158-17 (PMC5770554; doi:10.1128/mBio.02158-17)
Supplement: TABLE S1 [file mbo001183670st1.docx]

| **Table S1. Data collection and refinement statist** |  |
| --- | --- |
| **Data Collection** | |
| X-ray source | PXI/X06SA (SLS1) |
| Wavelength (Å) | 1 |
| Detector | PILATUS 6M |
| Temperature (K) | 100 |
| Space group | C 2 2 2_1_ |
| Cell dimentions |  |
| a; b; c (Å) | 110.56; 121.52; 112.76 |
| α; β; γ (◦) | 90.0; 90.0; 90.0 |
| Resolution (Å) | 2.65 (2.90-2.65) |
| Unique reflections | 21542 (4980) |
| Multiplicity | 2.9 (2.9) |
| Completeness (%) | 96.1 (95.2) |
| Rsym (%) | 4.0 (43.8) |
| Rmeas (%) | 4.9 (53.4) |
| Mean(I)/sd | 15.46 (2.38) |
| **Refinement** | |
| Resolution range (Å) | 81.78-2.65 |
| Number of reflections (working /test) | 20678 / 863 |
| Rcryst  (%) | 21.6 |
| Rfree (%) | 25.1 |
| Total number of atoms: |  |
| Protein | 3195 |
| Water | 17 |
| Ligand | 60 |
| Cobalt hexammine | 14 |
| Deviation from ideal geometry: |  |
| Bond lengths (Å) | 0.01 |
| Bond angles (°) | 1.32 |
| Bonded B’s (Å) | 2.5 |
| Ramachandran plot (%) |  |
| Most favoured regions | 92.9 |
| Additional allowed regions | 5.4 |
| Generously allowed regions | 1.1 |
| Disallowed regions | 0.5 |
