# Supplementary figures and images for: Molecular Insights into Function and Competitive Inhibition of Pseudomonas aeruginosa Multiple Virulence Factor Regulator
Source: mBio. 2018 Jan 16;9(1):e02158-17. doi: 10.1128/mBio.02158-17 (PMC5770554; doi:10.1128/mBio.02158-17)

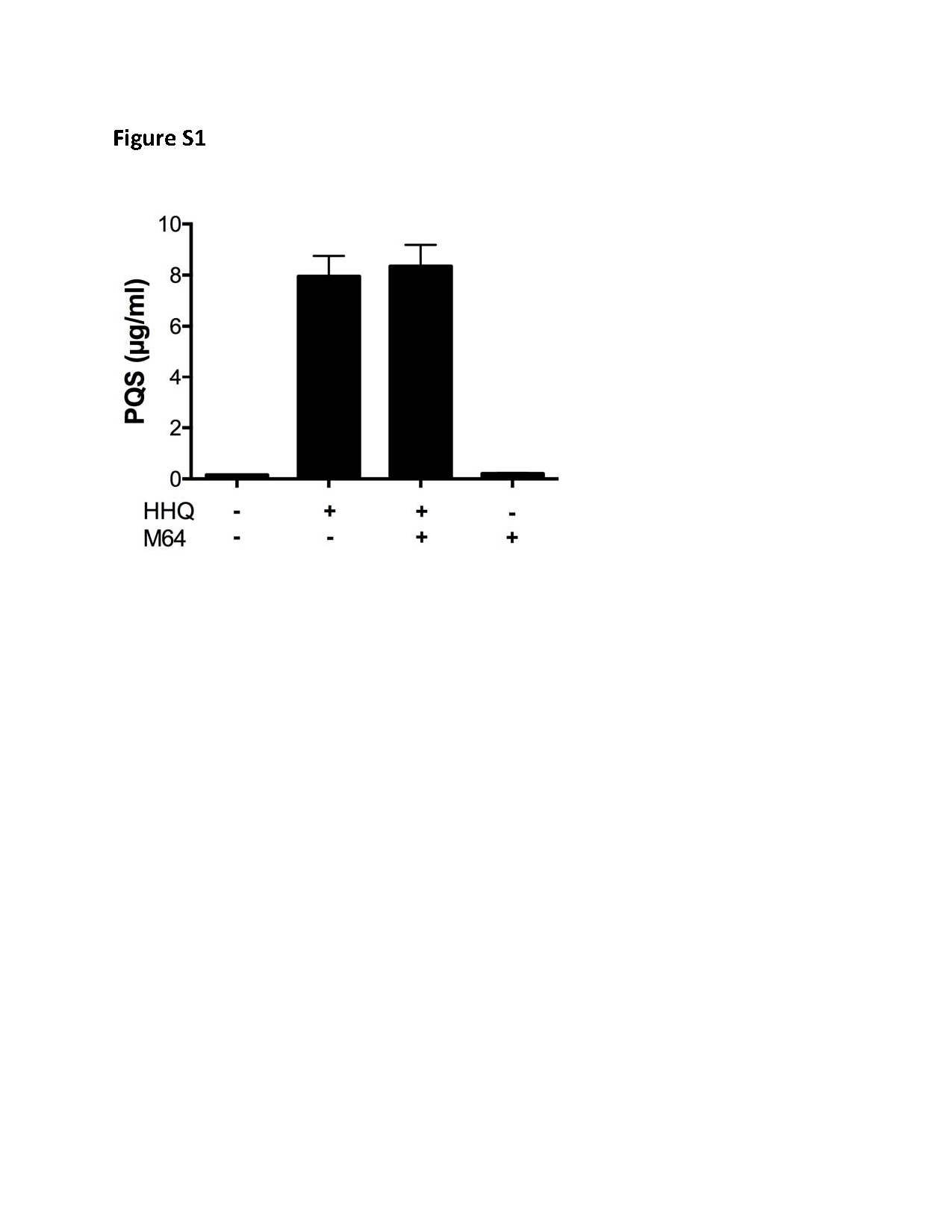

Supplement: FIG S1 [file mbo001183670sf1.tif]

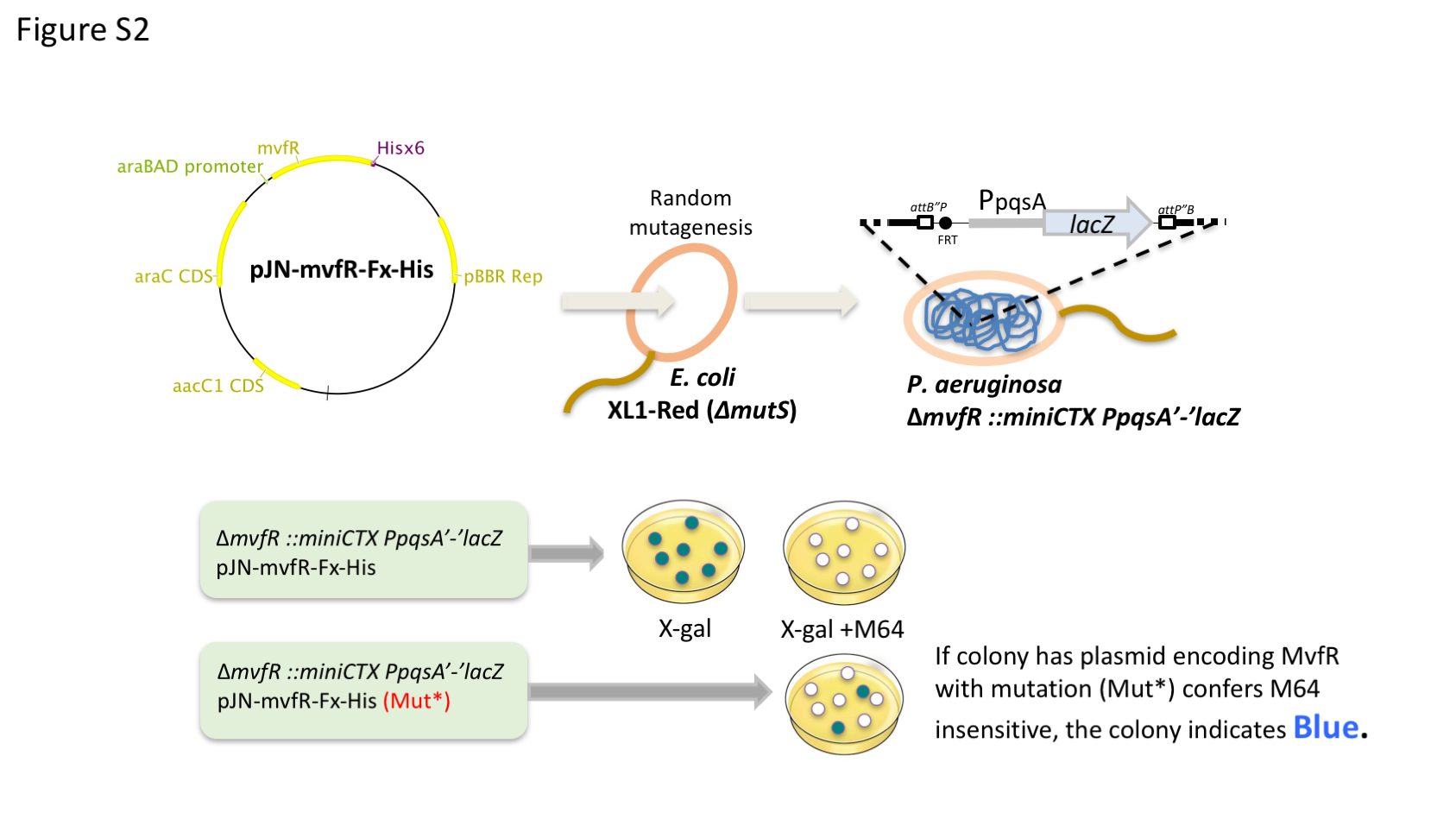

Supplement: FIG S2 [file mbo001183670sf2.tif]

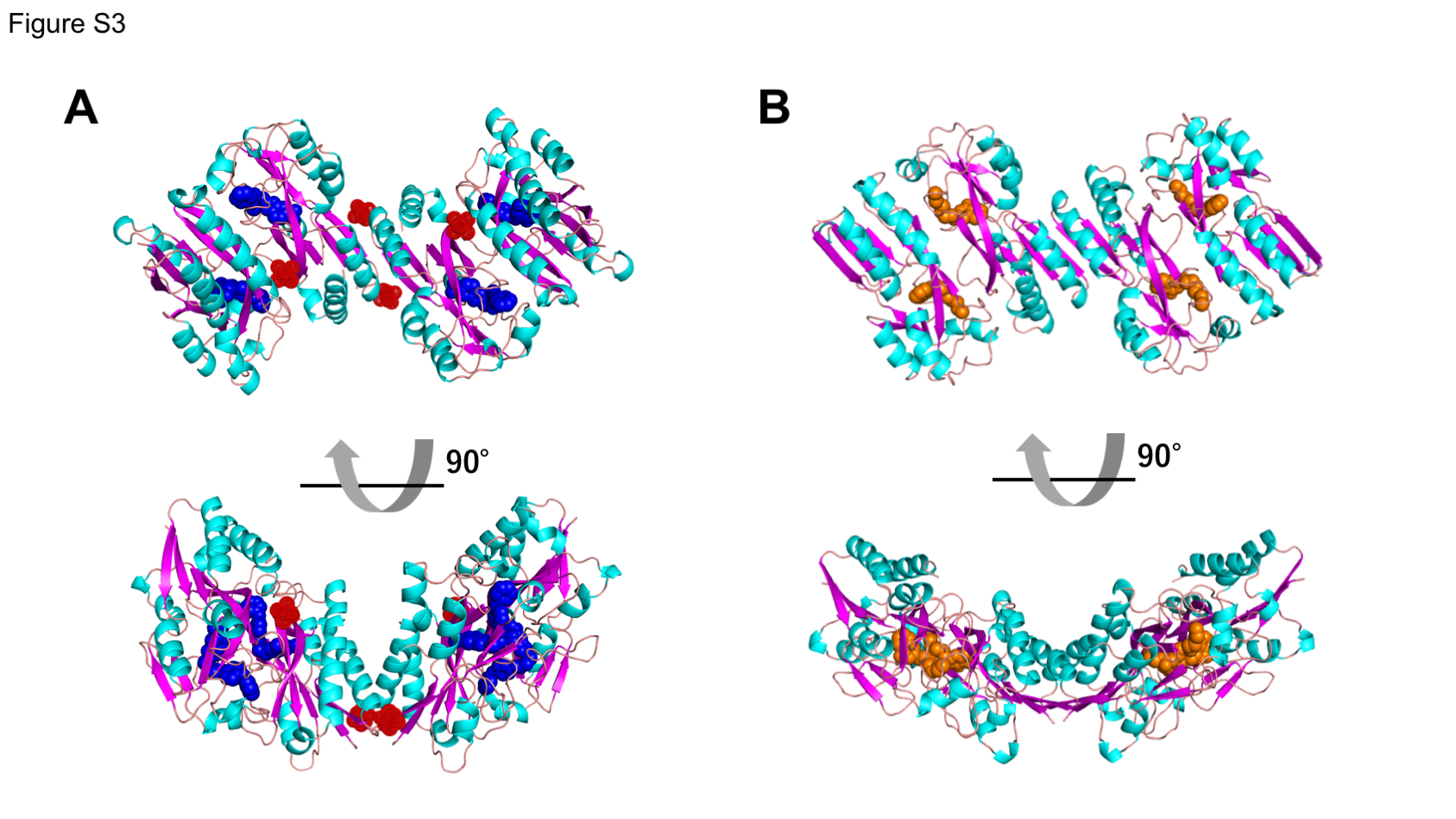

Supplement: FIG S3 [file mbo001183670sf3.tif]

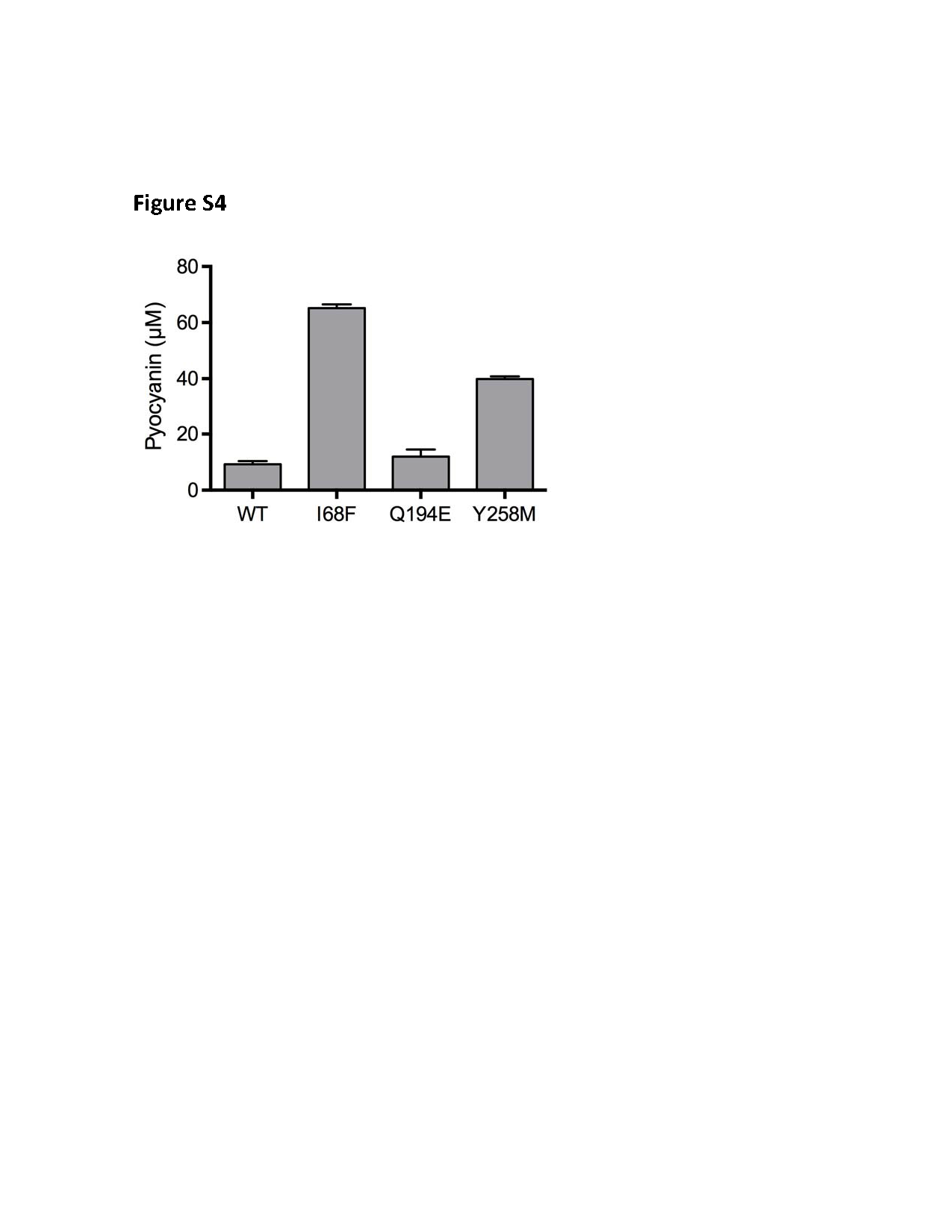

Supplement: FIG S4 [file mbo001183670sf4.tif]
