# Supplementary material for: Molecular Insights into Function and Competitive Inhibition of Pseudomonas aeruginosa Multiple Virulence Factor Regulator
Source: mBio. 2018 Jan 16;9(1):e02158-17. doi: 10.1128/mBio.02158-17 (PMC5770554; doi:10.1128/mBio.02158-17)
Supplement: TABLE S2 [file mbo001183670st2.docx]

| **Table S2. List of primers used in this study** | |
| --- | --- |
| **Name** | **Sequence (5' to 3')** |
| mvfR-Up-F | TGCAGGTCGACTCTAGAGTAGCTCGGCTCGCTCCACGACCGAA |
| mvfR-Up-R | CGCGTGCCGCTTCCCTTGAT |
| mvfR-Down-F | CAAGGGAAGCGGCACGCGAACGGTGCAGCGCCGCTCAGGC |
| mvfR-Down-R | TCGAGCTCGGTACCCGGGAGTTCTGCCTGCTCGGCGCGGAGGT |
| mvfR-F | CGTTTTTTTGGGCTAGCGGCCACCCAATAAAAGGAATAAGGGATGCC |
| His-FactorXa-mvfR-R | GATCCCCCGGGCTGCAGGTCAGTGGTGGTGGTGGTGGTGACGACCTTCGATTGCGGCGCGCTGGCGGTACGCGA |
| Q194E-1 | GCCAATTACCGGGAGATCAGCCTCGG |
| Q194E-2 | CCGAGGCTGATCTCCCGGTAATTGGC |
| Y258F-1 | CTCAGCGAACTCTTCGAACCGGGCGG |
| Y258F-2 | CCGCCCGGTTCGAAGAGTTCGCTGAG |
| Y258M-1 | CTCAGCGAACTCATGGAACCGGGCGG |
| Y258M-2 | CCGCCCGGTTCCATGAGTTCGCTGAG |
